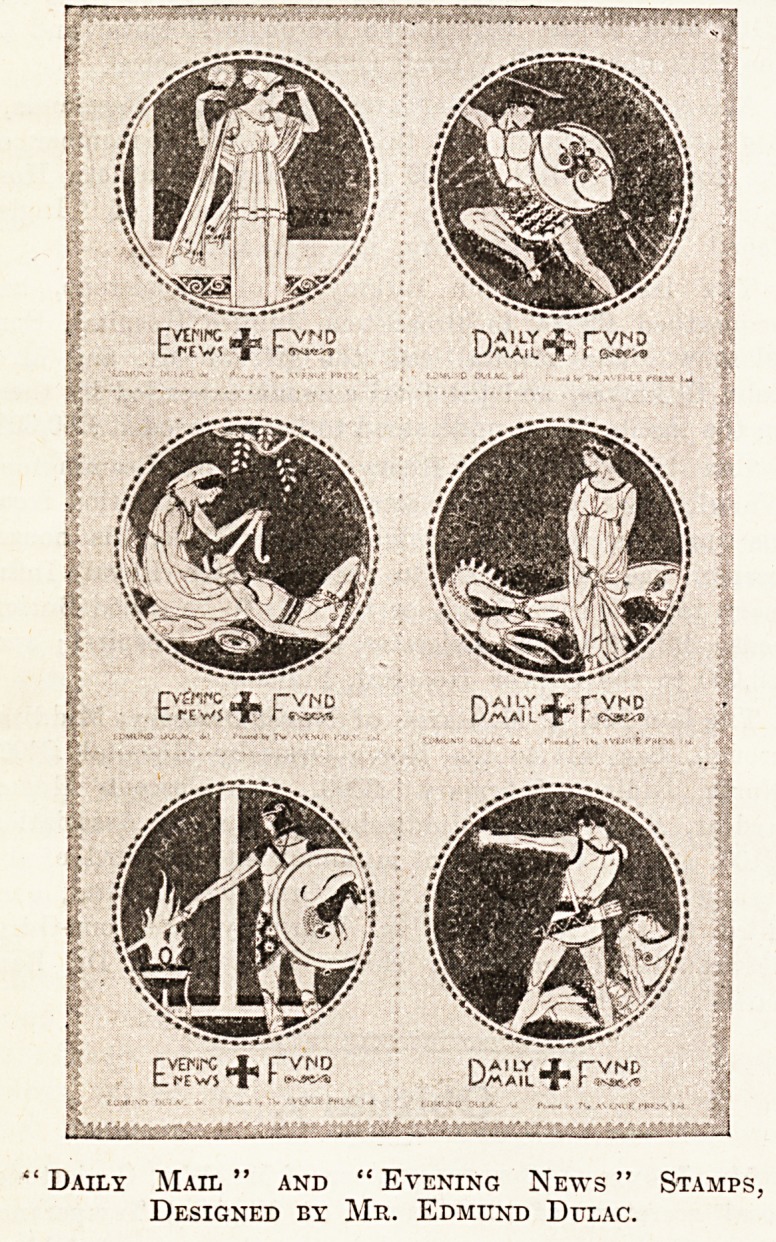# Art Stamps for Charitable Purposes

**Published:** 1915-01-16

**Authors:** 


### Art Stamps for Charitable Purposes.

For the purpose of popularising a children's Red Cross Fund, the *Daily Mail* and *Evening News* have devised a series of six art stamps from designs respectively by Frank Brangwyn, A.R.A., and Edmund Dulac. These, offered for sale at sixpence per set of six, and presumably intended for pasting on the *back* of envelopes, after the fashion of a seal, are shown in the accompanying illustrations, though the originals are in colours.

"Daily Mail" and "Evening News" Stamps, Designed by Mr. Frank Brangwyn, A.R.A.

"Daily Mail" and "Evening News" Stamps, Designed by Mr. Edmund Dulac.

As to the designs, readers will form their own opinions, though a comparison at once shows how adaptable, in the hands of a skilful draughtsman, a *tondo* is to arrest the eye. Certainly the two sets are in delightful contrast to each other. The stamps have a further interest for institutional readers in that they are but the latest attempt to employ philately for philanthropic purposes. The German—we might almost add the Continental—mind delights to garnish its letters and parcels with stamps and labels; and every exhibition, congress, and many institutions advertise themselves in this way. The English mind, however, regards even the postage stamp as a necessary evil; and the first popular criticism of Mr. Lloyd George's Insurance stamps will be still in the public mind. Stamps can be obtained from the Children's Red Cross Fund, Carmelite House, E.C.

## Figures and Tables

**Figure f1:**
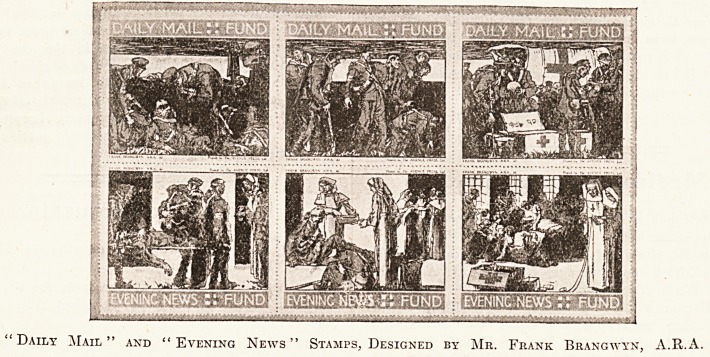


**Figure f2:**